# Emerging superbugs: The threat of Carbapenem Resistant Enterobacteriaceae

**DOI:** 10.3934/microbiol.2020012

**Published:** 2020-07-01

**Authors:** Le Thanh Dong, Helen V. Espinoza, J. Luis Espinoza

**Affiliations:** 1 Faculty of Medical Technology, Hanoi Medical University, Hanoi, Vietnam; 2 Faculty of Environmental Health, University of Washington, Seattle, WA, USA; 3 Faculty of Health Sciences, Kanazawa University, Kodatsuno 5-11-80, Kanazawa, 920-0942, Ishikawa, Japan

**Keywords:** multidrug-resistant bacteria, carbapenem, Super bugs, opportunistic infections, Carbapenem-resistant *Enterobacteriaceae*

## Abstract

Carbapenem-resistant Enterobacteriaceae (CRE) are gram-negative bacteria that are resistant to carbapenems, a group of antibiotics considered as the last-resource for the treatment of infections caused by multidrug-resistant bacteria. CRE constitutes a major threat to health care systems because infections caused by these pathogens are difficult to treat and are commonly associated with high mortality due to the limited availability of effective antibiotics. While infection prevention and timely detection are of vital importance to control CRE infections, developing new and effective anti-CRE therapies is also crucial. Accumulating evidence indicates that gut microbiota alteration (dysbiosis) is associated with an increased intestinal colonization with CRE and consequently with higher risk of developing CRE infections. Importantly, therapeutic interventions aimed to modify the gut microbiota composition via fecal microbiota transplantation (FMT) have been explored in various clinical settings with some of them showing promising results, although larger clinical trials are needed to confirm the efficacy of this strategy. Here, we highlight the challenges associated with the emergence of CRE infections.

## 1. Epidemiology and clinical aspects of CRE

The discovery of antibiotics is one of the most significant medical achievements of modern medicine as these agents have become a vital component of clinical practice; however, the emergence of antibiotic resistance, attributed to the overuse and misuse of these medications, constitutes a serious threat for global health. In the last two decades, complex resistant mechanisms have resulted in the emergence and worldwide propagation of multidrug-resistant organisms (MDRO) such as *Clostridioides difficile* (*C. difficile*), Methicillin-resistant Staphylococcus aureus (MRSA), Vancomycin-resistant Enterococci (VRE), Extended-spectrum β-lactamase (ESBLs) producing gram-negative bacteria, and Carbapenem-resistant Enterobacteriaceae (CRE), which have been detected in a variety of clinical settings [1].

Infections caused by CRE such as pneumonia and bloodstream infections are difficult to treat and are often associated with high mortality rates (crude mortality rates >70%) because carbapenems constitute the last line of antibiotic therapy against such infections. Although these organisms have primarily been recognized in health care settings, CRE have the potential to spread into the community. Indeed, the emergency of CRE is being increasingly reported in community settings [2]. Besides, the cost for treating infections linked to CRE represent an additional financial burden for healthcare systems, thus the spread of CRE in healthcare settings is both an important medical problem and a major global public health threat [3]. Due to these issues both the CDC and the WHO have listed CRE as ‘a critical priority threat that requires immediate action’.

Risk factors associated with increased CRE infections include, critical illness, comorbid conditions, chemotherapy for cancer, organ or stem cell transplantation, the use of invasive devices or mechanical ventilation, and previous use of broad-spectrum antimicrobials [2],[3]. Carbapenem resistance can be mediated by three major mechanisms: enzyme production, efflux pumps and porin mutation, with enzyme production (carbapenemase) being the main resistance mechanism. Carbapenemases hydrolyze most β-lactam molecules including carbapenems and are often encoded by mobile genetic elements that can be easily shared between bacteria, facilitating the rapid spread of resistance [4].

Although CRE isolates were first reported in the 1980s and various carbapenemases were identified across distinct species and strains in the 1990s [4], initially CRE were typically detected as hospital-acquired sporadic infections, which was followed by the emergence of single-hospital outbreaks and thereafter as multiple-hospitals outbreaks [5]. However, in the last decade, due in part to international travel and medical tourism, the incidence of CRE has markedly increased worldwide [6].

Currently, it is difficult to determine the global occurrence of CRE infections due to the unavailability of data on bacterial resistance in many regions of the world, however, epidemiologic studies suggest that the prevalence of CRE and the types of predominant carbapenemases are highly influenced by geographical factors [4],[5]. Clinically and epidemiologically important carbapenemases are classified into 3 classes: Ambler Class A, B, and D. In class A, *Klebsiella pneumoniae* carbapenemase (KPC) is the most prevalent and broadly extended carbapenemase, being endemic to regions in China, Israel, England, Italy, Romania, Greece, Brazil, Argentina, and Colombia [2],[3],[6]. Within Class B carbapenemases, also known as metallo-β-lactamases (MBLs), stand up the New Delhi MBL (NDM-1), which is endemic to the Indian subcontinent (Pakistan, India, Sri Lanka, and Bangladesh) but in recent years it has spread to the rest of the world as well [2],[5]. Class D carbapenemases include oxacillinase 48 (OXA-48)-producing CRE, causative of outbreaks in Mediterranean countries, although sporadic cases have been reported in other countries in association with recent travel to endemic regions [5],[6].

According to the most recent report of the CDC on antibiotic resistance in the US there were 13,100 estimated CRE infections in hospitalized patients in 2017 with an estimated of 1,100 deaths (CDC drug resistance report 2019). Similarly, a recent systematic review that included articles published from 2000 to 2016 reported that the incidence of CRE in the US was 0.3–2.93 infections per 100,000 person-years [7], indicating that the incidence of CRE is relatively low in comparison with other MDROs. For example, infections caused by MRSA [8] and *C. difficile* [9] are 8 and 65 times more common than CRE, respectively. Nevertheless, it must be noted that about 30% of CRE detected in the US are carbapenemase-producing CRE (CP-CRE) and considering that carbapenemase genes are often found on transferable plasmids, which can be easily shared between bacteria, there is a potentially risk of a wider spread of resistance. In addition, a study from the CDC reported that between 2 and 8% of surgical infections caused by *Escherichia coli* and *Klebsiella pneumoniae* isolates were CRE[10]. Furthermore, a population- and laboratory-based surveillance reported that 8% of the CRE were community acquired infections [11] suggesting the active presence of pathogen transmission in the community. This indicates that a failure to address the spread of CP-CRE could lead to further increases in CRE incidence.

Treatment of CRE depends on the site of infection, patients' clinical condition, isolated pathogen, and resistance profile [4],[5]. Given the complexity of these infections, treatment must be driven by *in vitro* susceptibility testing. Typically, treatment has been based on combination regimens with colistin coupled with other agents such as polymyxins, tigecycline, fosfomycin, or double carbapenem, although resistance to these agents has been reported [12]. New agents with activity against certain carbapenem-resistant pathogens such as the novel β-lactam/β-lactamase inhibitors aztreonam-avibactam, ceftazidime-avibactam, and meropenem-vaborbactam, have been approved for clinical use and other agents including plazomycin, cefiderocol, eravacycline, among others, are in various stages of development [4].

**Figure 1.The:**
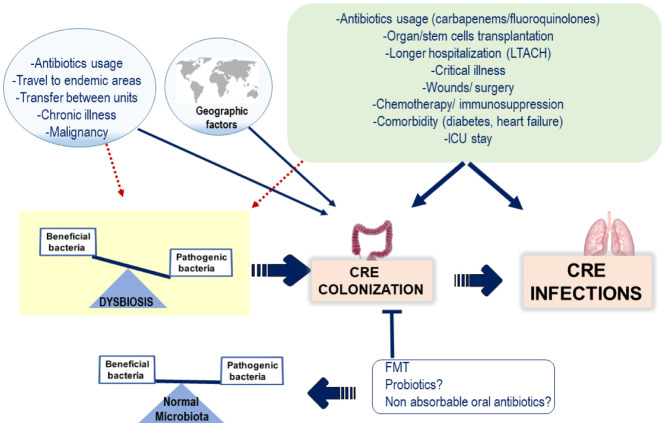
prevalence of CRE infections is influenced by geographic factors. Since intestinal colonization with CRE is strongly associated with CRE infections, risk factors for colonization with CRE are similar to those associated with CRE infections. Although distinct mechanisms may contribute to the increased risks of CRE acquisition linked to these factors, such as immunosuppression, tissue injury, malnutrition, etc.; it has become evident that most factors implicated in CRE acquisition induce intestinal dysbiosis and the disruption of the normal composition and function of the indigenous gut microbiota may result in the overgrowth of unwanted bacteria, including MDRO such as CRE. It has been proposed that restoring the microbiota composition via FMT and likely by using non-absorbable could contribute to eradicating intestinal CRE colonization and ultimately could reduce the incidence of CRE infections.

Another important aspect in CRE is the rapid emergence of asymptomatic infections in the form of bacterial colonization, which has been detected not only in hospitalized patients but also in the community setting [6]. Digestive tract carriage with CRE has been associated with high rates of subsequent infection, especially in high risk patients. For example, an increased relative abundance of CRE in the gut microbiota of patients hospitalized in long-term acute care hospitals (LTACH) was associated with CRE-induced bacteremia [13]. Risk factors for colonization with CRE are similar to those associated with other MDRO, such as prior antibiotic usage, healthcare exposure, advanced age and chronic comorbid conditions (diabetes mellitus, heart disease, and kidney failure)[2]–[5]. High prevalence of intestinal carriage of CRE has been also detected in cancer patients receiving chemotherapy [14] as well as traveling to endemic areas [11] (Figure 1).

A recent study from Vietnam that included more than 2,200 patients admitted to 63 different wards at 12 hospitals in various parts of the country reported that a mean of 52% of patients admitted to hospitals throughout Vietnam were colonized with CRE. Importantly, the mean CRE colonization rates increased from 13% on the day of admission to 89% at Day 15 of hospitalization, indicating that there is an epidemic spread of CRE in Vietnamese hospitals with rapid transmissions in hospitalized patients. Interestingly, a sub-analysis of 328 new-born children in neonatal intensive units found a significant association between CRE colonization, hospital-acquired infection and mortality (odds ratio 5.5, p < 0.01) [15].

## 2. Fecal microbiota transplantation and CRE

Accumulating evidence suggest that the composition of gut microbiota plays a critical role in determining susceptibility to CRE carriage and ultimately infection. It is well-known that intestinal dysbiosis may lead to an overgrowth of pathogenic microorganisms, including MDRO. For example, infection with *C. difficile* frequently develop in the presence of an altered gut microbiota following antibiotic administration[16] and microbiota restoration using fecal microbiota transplantation (FMT) effectively eradicates recurrent *C. difficile* infection [17].

In line with these observations, it is reasonable to assume that restoration of normal gut microbiota may contribute to the eradication of CRE carriage. Indeed, several studies have found relationship between dysbiosis and the spread of CRE as demonstrated by a recent study linking intestinal dysbiosis with intestinal colonization with CRE and ultimately a higher prevalence of bacteremia in hospitalized patients [18], a phenomenon that is particularly frequent in LTACH [13].

Several studies have tested the efficacy of FMT in the eradication of CRE colonization. For example, in a study conducted in France, eight patients with digestive tract colonization by CRE or VRE, six carrying CRE and two colonized by VRE underwent FMT and by one month after the procedure, two patients were free from CRE carriage, and another patient was free from VRE after three months [19]. Similarly, three months after undergoing FMT, 50% of patients were free of intestinal CRE colonization, according to another study from France [20]. A randomized, open-label trial involving 39 non-immunocompromised adult patients reported that non-absorbable antibiotics followed by FMT, decreased ESBL-E/CPE carriage compared with controls (41% vs 29%, OR for decolonization success of 1.7, 95% CI 0.4–6.4) [21]. In a prospective study involving patients with hematological disorders and carriers of various CRE strains 15 out of 20 patients experienced complete decolonization after undergoing FMT and despite all patients enrolled in this study being immunosuppressed, there were no severe adverse events associated with FMT [22].

Given the apparent effectivity of FMT for the eradication of MDR microorganisms, including CRE, clinicians are increasingly aware about this therapy, however, in the setting of CRE colonization, there are several uncertainties about the optimal approach towards eradication of these pathogens. For example, the optimal route of application (nasogastric, colonoscopy or capsule), has not been defined and other important aspects such as the preconditioning protocols, the age of patients and donors, as well as the dosage and frequency of administration are yet to be established.

In addition, since many intestinal carriers of CRE are immunodeficient patients, it is important to determine the impact of immunosuppression on the efficacy of FMT and vice versa, the safety of this procedure in immunocompromised patients need to be determined as there are concerns on possible septic complications, however, despite the number of enrolled patients in this study was small, FMT appears to be safe in immunocompromised patients [22].

## 3. Conclusions and future directions

CRE have become endemic in several health care institutions across the world with the potential spread to community settings and while new antibiotics with activity against CRE are urgently needed, the role of antimicrobial stewardship is essential for the appropriate and rational use of existing antibiotics. In addition, while the proper use of antibiotics in the clinical setting is vital, the extensive use of these agents outside medical settings (animal husbandry, fish farming, etc.) must be rigorously regulated since the inappropriate use of antibiotics contributes to the selection and spread of antibiotic-resistant strains. Given the limited antimicrobial therapies available to combat CRE infections, so far the most reliable resource to control the spread of these pathogens is prevention. Therefore, understanding the local and global epidemiology of CRE is fundamental for designing prevention strategies. In addition, screening, and prompt detection of suspected isolates coupled with other measures useful for other resistant microorganisms, including the use of barrier precautions and isolation, aggressive environmental cleaning is crucial for infection control.

Finally, it is currently unknown the potential impact that the ongoing COVID-19 pandemic will have on the incidence of CRE infections. As health care is being focused in combating the novel coronavirus and the overstretched health systems are unable to operate effectively, it is plausible that interruptions in health services and supplies disruptions caused by the COVID-19 pandemic could result in the undetected propagation of CRE in the health care and community settings, a phenomenon that has been observed in previous epidemics. For example, reduction in access to healthcare services during the 2014–2015 Ebola outbreak exacerbated malaria, HIV/AIDS, and tuberculosis mortality rates in the affected countries [23]. On the other hand, it is plausible that the stricter infection control measures being currently applied in the health care setting to prevent covid-19 infection among health care providers may contribute to decreasing the spread of other nosocomial pathogens including CRE. Indeed, lessons learned from this pandemic, in terms of virus infection prevention and the experience of fighting against an infectious agent for which no specific treatment is available, will likely change the future of health care, which could result in a reduction of MDRO infections.
